# Supplementary material for: Microbial Recycling of Bioplastics via Mixed-Culture Fermentation of Hydrolyzed Polyhydroxyalkanoates into Carboxylates
Source: Materials (Basel). 2023 Mar 28;16(7):2693. doi: 10.3390/ma16072693 (PMC10096456; doi:10.3390/ma16072693)
Supplement: Supplementary file 1 [file materials-16-02693-s001.zip › materials-2216367-supplementary.pdf]

Supplementary Materials

Microbial Recycling of Bioplastics via Mixed-Culture Fermentation of Hydrolyzed Polyhydroxyalkanoates into Carboxylates

Yong Jin [1], Kasper D. de Leeuw [1,2], and David P. B. T. B. Strik [1,*]

[1] Environmental Technology, Wageningen University & Research, 6708 WG Wageningen, The Netherlands; yong.jin@wur.nl (Y.J.); kasper.deleeuw@wur.nl (K.D.d.L.)
[2] ChainCraft B.V., 1043 AP Amsterdam, The Netherlands
* Correspondence: david.strik@wur.nl

Table S1. Typical material properties of PHBV [1].

Item	Data	Test Standard
Specific gravity	1.25	ISO 1183-3:1999
Melt flow index	12-25	ISO 1133:1997, g/10 min, 190 °C, 2.16 kg
Yield stress (MPa)	31-36	ISO 527-1:1993
Tensile strength (MPa)	39	ISO 527-1:1993
Elongation at break (%)	3.8	ISO 527-1:1993
Young's modulus (MPa)	160–2100	ISO 527-1:1993
Flexural modulus (MPa)	2200–2900	ISO 178:1993
Vicat softening temperature (°C)	166	ISO 306:1994
Izod impact (J/m)	55–60	ISO 180:2000
Heat deflection temperature (°C)	157–165	ISO 75-2:2003
DSC melting point (°C)	175–180	ISO 11357-3:1999

Table S2. Compositions of stock solutions used to prepare medium for mixed culture fermentation of PHBV.

Compound	Concentration (g/L)	Dilution Factor
	Stock solution I-Minerals	
$NH_4H_2PO_4$	180	50×
$MgCl_2·6H_2O$	16.5	
$MgSO_4·7H_2O$	10	
	Stock solution II-Minerals	
$CaCl_2·2H_2O$	10	50×
KCl	7.5	
	Trace metals	
$FeCl_2·4H_2O$	30.0	2000×
$MnCl_2·4H_2O$	0.6	
H_3BO_3	6.0	
$CoCl_2·6H_2O$	4.0	
$CuCl_2·H_2O$	0.2	
$NiCl_2·6H_2O$	0.4	
$ZnSO_4·7H_2O$	2.0	
$Na_2MoO_4·2H_2O$	0.6	
Na_2SeO_3	0.2	
EDTA	12.4	
	Vitamins	
Biotin	0.106	1000×
Folic acid	0.005	
Pyridoxal-HCl	0.0025	
Lipoic acid	0.015	
Riboflavin	0.0125	
Thiamine-HCl	0.266	
Ca-D-pantothenate	0.413	
Cyanocobalamin	0.0125	
P-aminobenzoic acid	0.0125	
Nicotinic acid	0.0125	
	Supplements	
Yeast extract	0.1	
HCl	1 M	
KOH	4 M	

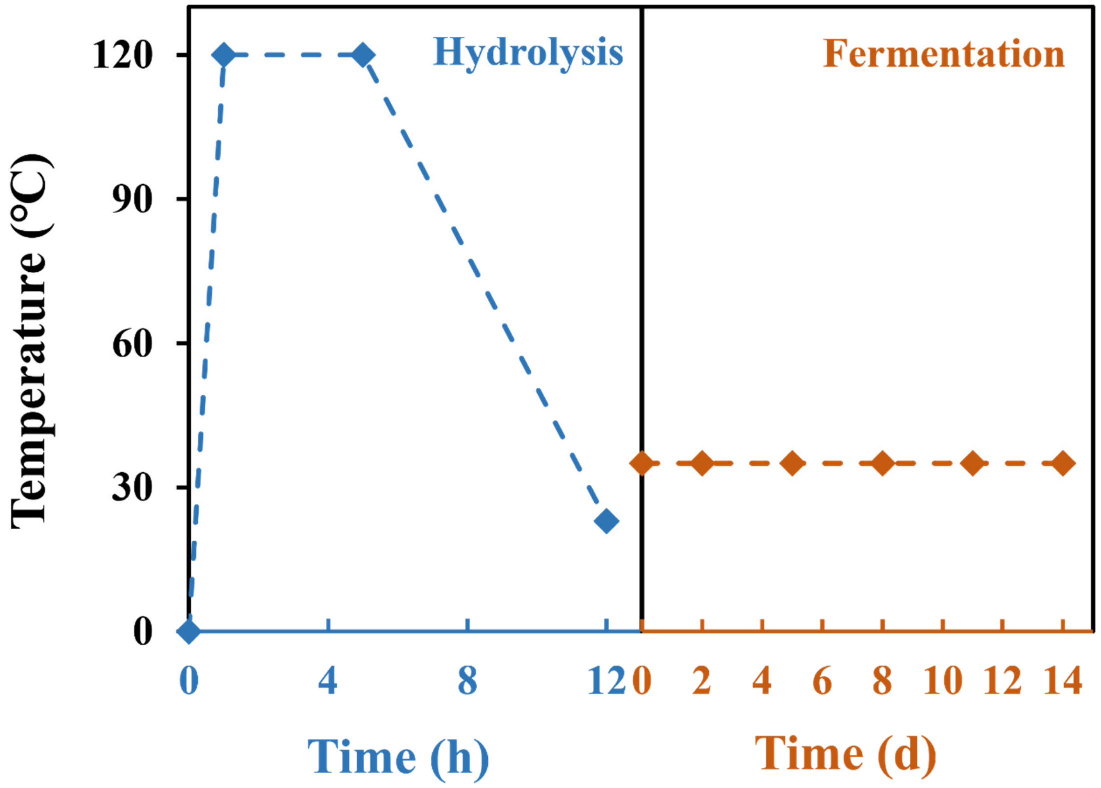

Figure S1. Temperature changes in thermal hydrolysis and fermentation process.

Table S3. Total Chemical Oxygen Demand (TCOD).

Material	Test	COD (g/kg)	Average COD (g/kg)
PHBV (2% HV)	A	1570	1588
	B	1605	

a. To determine the chemical oxygen demand z. B. according to DIN 38409/German standard procedure H41. b. Digestion in the aluminum heating block with precise temperature control. c. Heat samples to 148 °C in less than 10 min.

Calculations S1. Estimated COD recovery for a potential PHA to PHA recycling process

The PHA to PHA recovery percentage was calculated according:

$$\boldsymbol{PHA_{recovery}} = \left(\boldsymbol{Yield_{PHA\ hydrolysis}} - \boldsymbol{Yield_{biomass}}\right) * \boldsymbol{Yield_{PHA\ from\ VFA}} * \mathbf{100\%}\ [\%] \qquad (S1)$$

where by the following targets or experimental findings were used:
Yield $_{\text{crotonate+3-HB from PHA}}$ = 1 [g corotonate+3HB-COD/g PHA-COD];
Yield $_{\text{PHA from VFA}}$: 0.61-0.68 [g PHA-COD/g VFA-COD] could be produced in a synthetic or nutrient-poor stream [2];
Yield $_{\text{biomass}}$: a typical 0.05 biomass yield for microbial growth [3] [g biomass-COD/g VFA-COD];
So the overall recovery percentage of PHA was estimated to be 58% (= (1-0.05)*0.61*100%) to 65% (= (1-0.05)*0.68*100%).

Calculations S2. The overall yields of VFAs from PHA hydrolysis

$$\boldsymbol{Yield_{VFA}} = \frac{\boldsymbol{C_{VFA}}}{\boldsymbol{C_{original\ PHA}}} * \left(\boldsymbol{COD_{hydrolysates}}/\boldsymbol{COD_{start\ of\ fermentation}}\right)\ \ [g\ VFA/g\ PHA] \quad \text{(S2)}$$

COD $_{\text{hydrolysates}}$: 16.3 [g COD/L];

COD $_{\text{start of fermentation}}$: 4 [g COD/L];

So the Yield acetate was 0.07 = (1.71/100)*(16.3/4) [g acetate/g PHA], and Yield butyrate was 0.049 = (1.20/100)*(16.3/4) [g butyrate/g PHA].

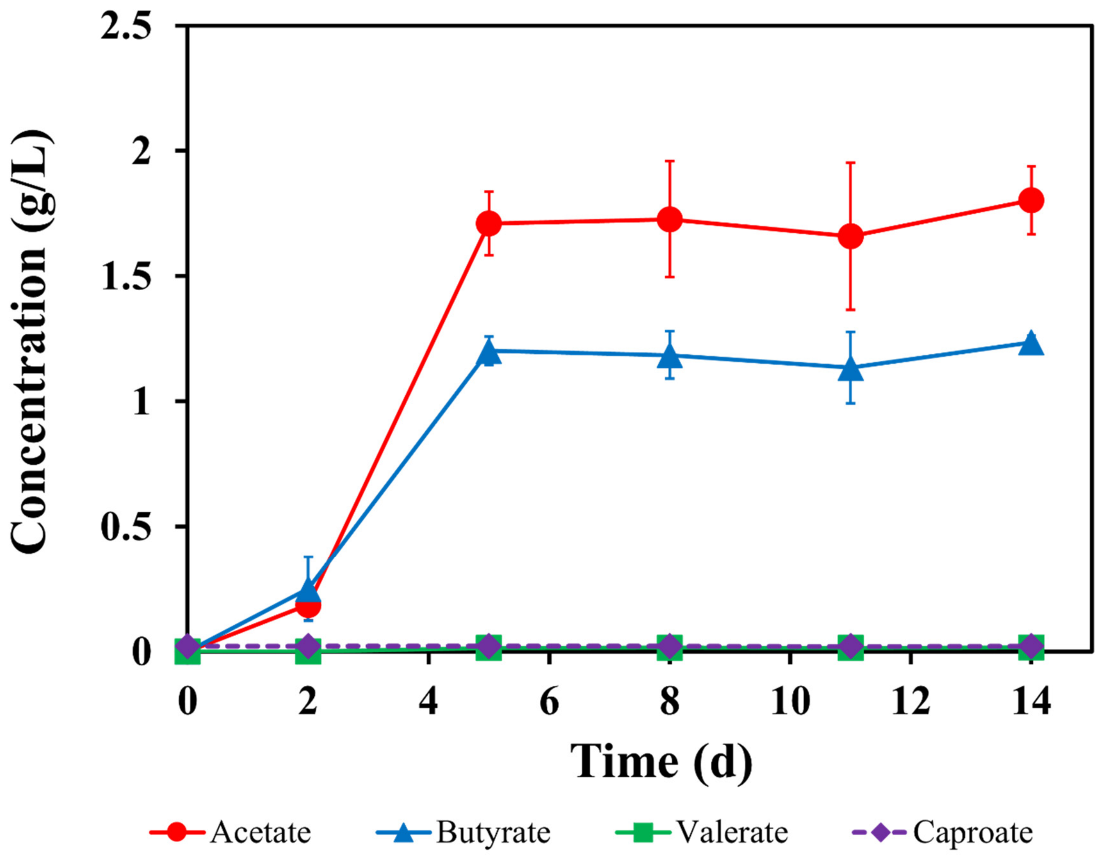

Figure S2. Volatile fatty acids distribution during mixed culture fermentation of PHA. The visible error bars represent the standard deviation of the triplicate experiments.

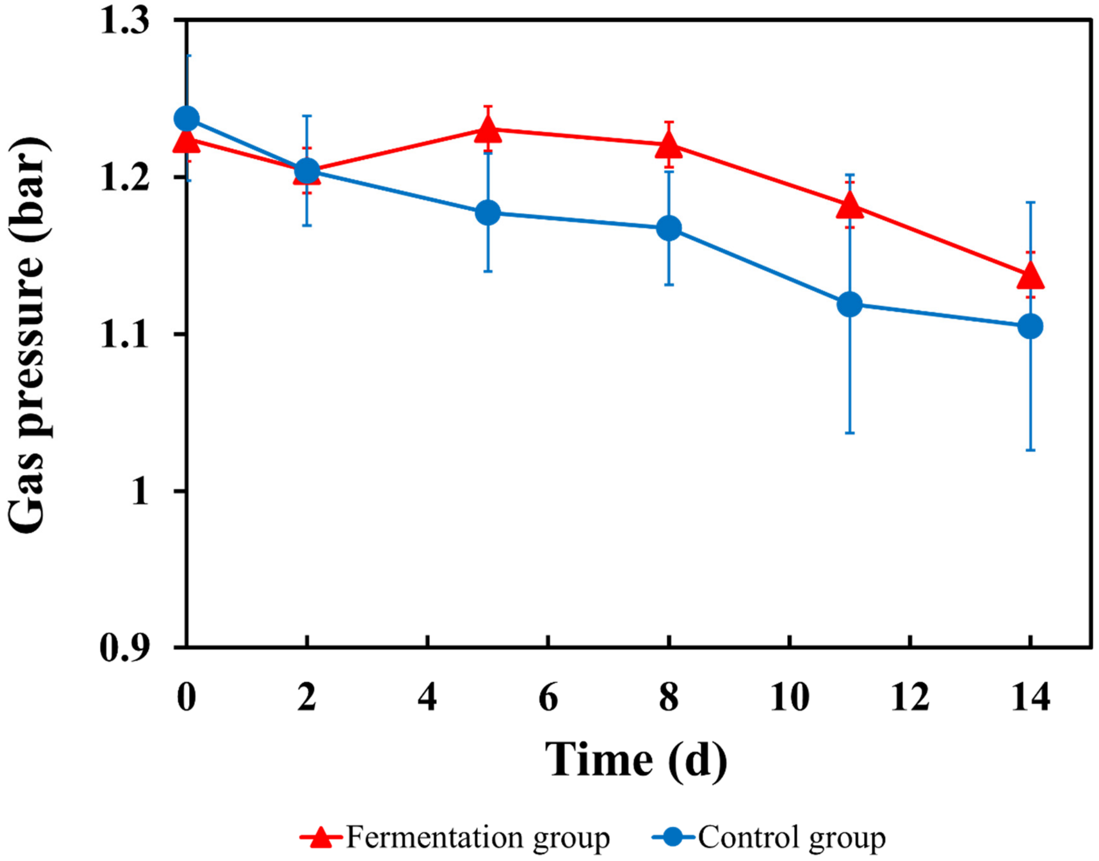

Figure S3. Gas pressure measured during mixed culture fermentation of PHA. The visible error bars represent the standard deviation of the triplicate experiments.

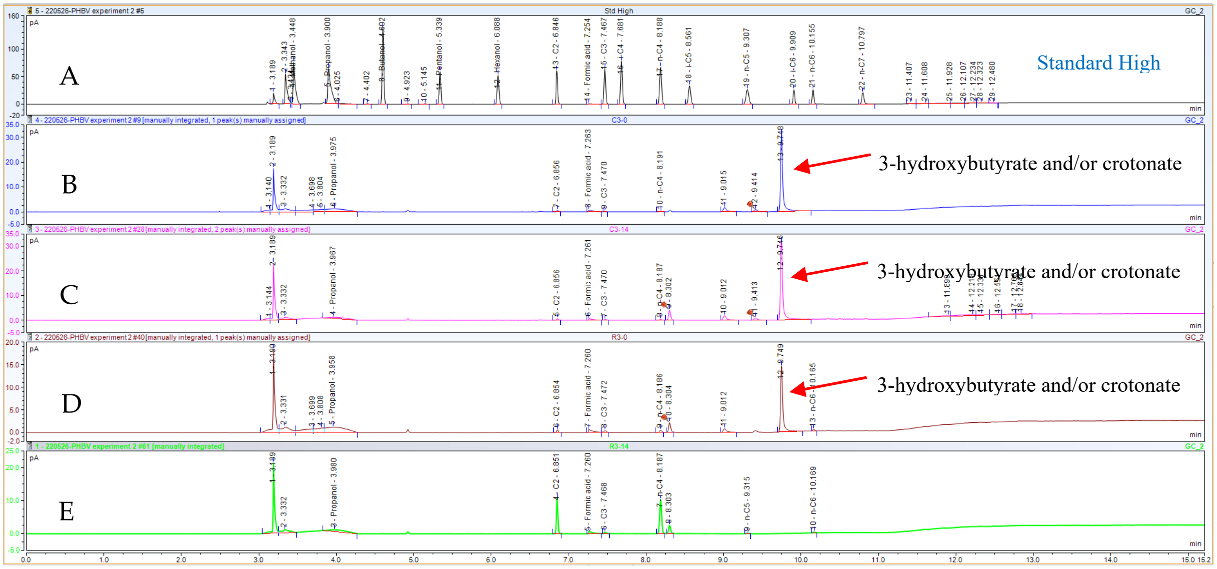

Figure S4. Carboxylates measurement in a gas chromatograph (GC) of selected samples. **Note: A** is the standard curve in high-concentration solution; **B** is the curve of the start experiment of the control group; **C** is the curve of the end experiment of the control group; **D** is the curve of the start experiment of the fermentation group; **E** is the curve of the end experiment of fermentation group.

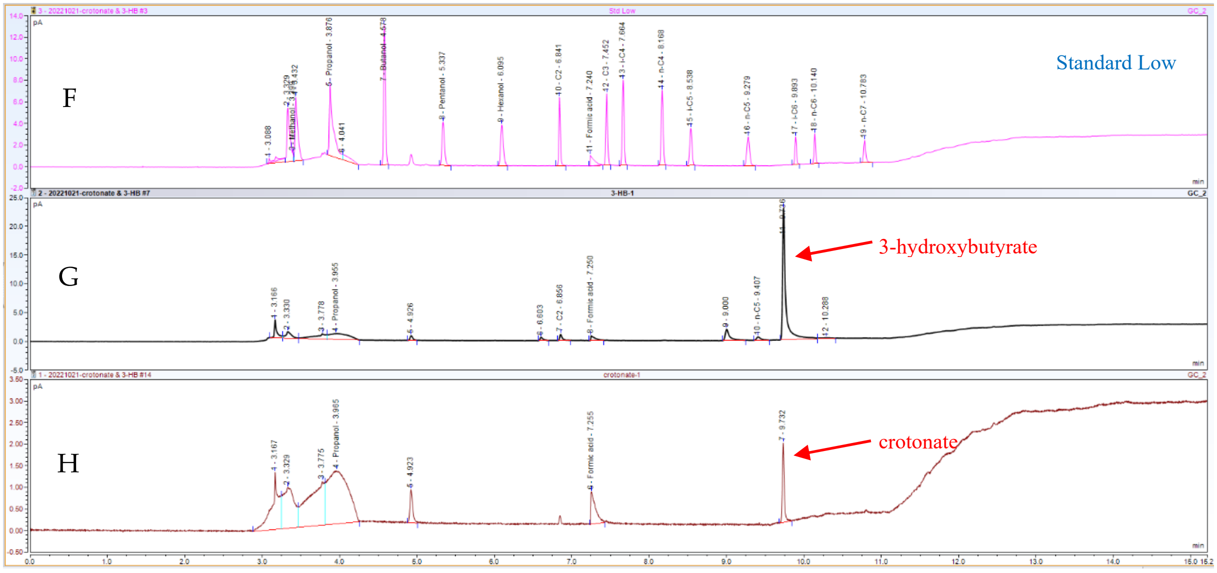

Figure S5. Crotonate and 3-hydroxybutyrate (3-HB) measurement in a gas chromatograph (GC). **Note: F** is the standard curve in low-concentration solution; **G** is the curve of 500 mg/L 3-hydroxybutyrate; **H** is the curve of 10 mg/L crotonate. **G** and **H** are selected from duplicate samples.

Compared with the retention time of the peaks of quantitively measured crotonate and 3-hydroxybutyrate in groups **G** and **H**, here the peaks showed with red rows in groups **B-D** indicating that there were some crotonate and/or 3-hydroxybutyrate produced from PHBV hydrolysis, and in group **E**, no peak at the same time as showed in **B** to **D**, this can qualitatively identify the present crotonate and/or 3-hydroxybutyrate and the conversion of them into acetate and butyrate (both peaks of these two carboxylates increased apparently).

Table S4. Compositions of gas in headspace measured at the end of fermentation.

Group	O_2 (%)	N_2 (%)	CO_2 (%)	H_2 (%)	CH_4 (%)
Fermentation 1	0.22	84.7	13.7	0.010	0
Fermentation 2	0.21	86.3	12.7	0.588	0
Fermentation 3	0.23	85.7	13.0	n.a.	0
Control 1	0.17	96.9	10.1	n.a.	0
Control 2	0.17	90.4	8.96	n.a.	0
Control 3	0.21	90.5	8.66	n.a.	0

Note: n.a. means ‘not available’.

References

1. García-Depraect, O.; Lebrero, R.; Rodriguez-Vega, S.; Bordel, S.; Santos-Beneit, F.; Martínez-Mendoza, L.J.; Aragão Börner, R.; Börner, T.; Muñoz, R. Biodegradation of bioplastics under aerobic and anaerobic aqueous conditions: Kinetics, carbon fate and particle size effect. *Bioresour. Technol.* **2022**, *344*, 126265. https://doi.org/10.1016/j.biortech.2021.126265.
2. Estévez-Alonso, Á.; Pei, R.; van Loosdrecht, M.C.M.; Kleerebezem, R.; Werker, A. Scaling-up microbial community-based polyhydroxyalkanoate production: status and challenges. *Bioresour. Technol.* **2021**, *327*, 124790. https://doi.org/10.1016/j.biortech.2021.124790.
3. Geleti, D.; Hailemariam, M.; Mengistu, A.K.; Tolera, A. Biomass yield potential and nutritive value of selected Alfalfa (*Medicago sativa* L.) cultivars grown under tepid to cool sub-moist agro-ecology of Ethiopia. *E3 J. Agric. Res. Dev.* **2014**, *4*, 007–014.
